# Aphids acquired symbiotic genes via lateral gene transfer

**DOI:** 10.1186/1741-7007-7-12

**Published:** 2009-03-10

**Authors:** Naruo Nikoh, Atsushi Nakabachi

**Affiliations:** 1 Division of Natural Sciences, The Open University of Japan, Chiba, Japan; 2 Advanced Science Institute, RIKEN, Saitama, Japan

## Abstract

**Background:**

Aphids possess bacteriocytes, which are cells specifically differentiated to harbour the obligate mutualist *Buchnera aphidicola *(γ-Proteobacteria). *Buchnera *has lost many of the genes that appear to be essential for bacterial life. From the bacteriocyte of the pea aphid *Acyrthosiphon pisum*, we previously identified two clusters of expressed sequence tags that display similarity only to bacterial genes. Southern blot analysis demonstrated that they are encoded in the aphid genome. In this study, in order to assess the possibility of lateral gene transfer, we determined the full-length sequences of these transcripts, and performed detailed structural and phylogenetic analyses. We further examined their expression levels in the bacteriocyte using real-time quantitative RT-PCR.

**Results:**

Sequence similarity searches demonstrated that these fully sequenced transcripts are significantly similar to the bacterial genes *ldcA *(product, LD-carboxypeptidase) and *rlpA *(product, rare lipoprotein A), respectively. *Buchnera *lacks these genes, whereas many other bacteria, including *Escherichia coli*, a close relative of *Buchnera*, possess both *ldcA *and *rlpA*. Molecular phylogenetic analysis clearly demonstrated that the aphid *ldcA *was derived from a rickettsial bacterium closely related to the extant *Wolbachia *spp. (α-Proteobacteria, Rickettsiales), which are intracellular symbionts of various lineages of arthropods. The evolutionary origin of *rlpA *was not fully resolved, but it was clearly demonstrated that its double-ψ β-barrel domain is of bacterial origin. Real-time quantitative RT-PCR demonstrated that *ldcA *and *rlpA *are expressed 11.6 and 154-fold higher in the bacteriocyte than in the whole body, respectively. LdcA is an enzyme required for recycling murein (peptidoglycan), which is a component of the bacterial cell wall. As *Buchnera *possesses a cell wall composed of murein but lacks *ldcA*, a high level of expression of the aphid *ldcA *in the bacteriocyte may be essential to maintain *Buchnera*. Although the function of RlpA is not well known, conspicuous up-regulation of the aphid *rlpA *in the bacteriocyte implies that this gene is also essential for *Buchnera*.

**Conclusion:**

In this study, we obtained several lines of evidence indicating that aphids acquired genes from bacteria via lateral gene transfer and that these genes are used to maintain the obligately mutualistic bacterium, *Buchnera*.

## Background

Aphids are hemipteran insects that have close associations with various lineages of microorganisms. Most aphid species harbour the obligate mutualist (usually called primary symbiont), *Buchnera aphidicola *(γ-Proteobacteria), within the cytoplasm of specialized cells called bacteriocytes [1-4]. Since the initial infection more than 100 million years ago [5], *Buchnera *have been subjected to strict vertical transmission through host generations, and the mutualism between *Buchnera *and their host has evolved to the point that neither can reproduce in the absence of the other. *Buchnera *cannot proliferate outside bacteriocytes and, when deprived of *Buchnera*, the host insects suffer retarded growth and sterility, as they are obligately dependent on *Buchnera *for the supply of essential nutrients they cannot synthesize, and which are scarce in their diet of phloem sap [4,6-9]. During the process of co-evolution with the host, *Buchnera *has lost a number of genes that appear to be essential for bacterial existence (the genomes of *Buchnera *range from 420 to 650 kb in size and contain 400 to 600 genes; [10-13]); this raises the question of how *Buchnera *survive within the host bacteriocyte. One of several possible explanations for the absence of these genes is that some genes were transferred from the genome of an ancestor of *Buchnera *to the genome of an aphid ancestor and are now expressed under the control of the host nucleus. Such lateral gene transfer (LGT) should take place in the germ line for the transferred gene to be inherited through the generations of the recipient. During most of their life stages, *Buchnera *are confined within bacteriocytes, which are segregated from germ cells; however, the symbionts are freed from the maternal bacteriocytes before being transmitted to the next generation. In cases of parthenogenetic reproduction, *Buchnera *cells are transferred into the parthenogenetic blastoderm-stage embryos; *Buchnera *are localized proximal to the host germ cells during early development of the host. Moreover, in cases of sexual reproduction, *Buchnera *enter sexual eggs at the pre-cellularization stage; at this stage, there are no membranous barriers between *Buchnera *and the germ lines [14,15]. Such localization of *Buchnera *cells proximal to host germ lines might provide opportunities for the LGT from *Buchnera *into the germ lines.

In addition to *Buchnera*, a number of aphid strains harbour other maternally transmitted intracellular bacteria, such as *Rickettsia *(α-Proteobacteria), *Spiroplasma *(Mollicutes), and various γ-proteobacterial microbes, including *Hamiltonella defensa, Regiella insecticola, Serratia symbiotica*, and *Arsenophonus *species [16-28]. These 'secondary symbionts' are often shared between divergent insect lineages. For example, *Hamiltonella *and *Arsenophonus *are observed in scattered strains and species of aphids, psyllids, whiteflies and planthoppers [22,24,29]. *Wolbachia *lineages (α-Proteobacteria, Rickettsiales) are observed in a wide variety of arthropods [30-32], though only one case of infection has been reported in aphids [23]. These suggest that secondary symbionts undergo horizontal transfer among matrilines within and between species. They are also transmitted vertically [16-28], but this appears to be achieved in a less tightly controlled manner in comparison to the case of *Buchnera *[1]. Whereas *Buchnera *exist as passive symbionts within their hosts, which in turn have evolved mechanisms to maintain and transmit the *Buchnera *[14,15,33], secondary symbionts overcome host immune responses and invade various types of host cells, including germ cells [1,18,21,23,25,27,34]. Thus, there are likely to have been frequent opportunities for aphids to acquire genomic fragments from these symbiotic bacteria during evolution.

We previously performed transcriptome analysis of the bacteriocyte of the pea aphid *Acyrthosiphon pisum*, to elucidate the host mechanisms required to maintain *Buchnera *[33]. This study identified a number of aphid genes that are highly expressed in the bacteriocyte. Among them, two genes (corresponding to the cDNA clusters R2C00193 and R2C00214, which consist of 10 and four expressed sequence tags, respectively) exhibited similarity only to prokaryotic genes, and not to those of extant *Buchnera *lineages. Southern blot analysis confirmed that they are encoded in the aphid genome.

In the present study, we show the detailed analysis of the phylogenetic positions, domain structures, and expression profiles of these genes, thus revealing their evolutionary history and functional roles.

## Results

### Full-length sequencing of cDNA clones

**Figure 1:**
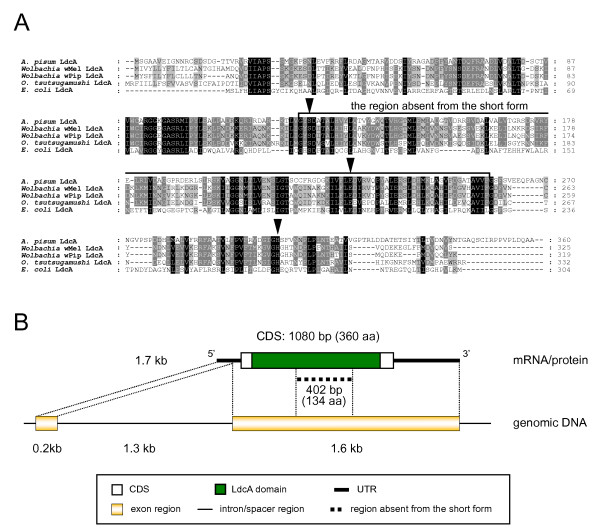
**Structure of the aphid LdcA**. (A) ClustalX alignment of amino acid sequences of LdcAs. Residues conserved in all lineages, four lineages, and three lineages are shaded black, dark gray, and light gray, respectively. Arrowheads indicate the residues required for LdcA activity. The long form of the aphid LdcA is used for the alignment. (B) Domain structure of the aphid LdcA protein and structures of the corresponding mRNA and genomic DNA.

**Figure 2:**
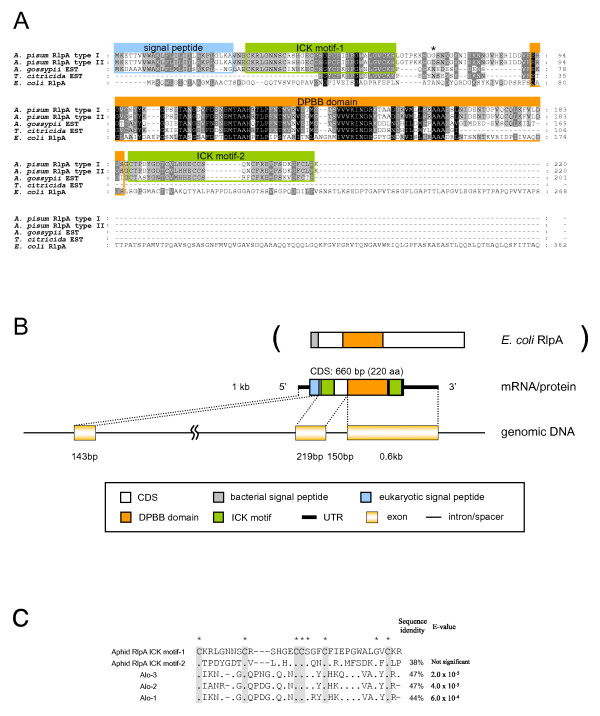
**Structure of the aphid RlpA**. (A) ClustalX alignment of amino acid sequences of RlpAs. Residues conserved in all lineages, four lineages, and three lineages are shaded black, dark gray, and light gray, respectively. Residues contributing to the domain structures are boxed. A residue that is different between type I and type II of *A. pisum *RlpA is denoted with an asterisk. (B) Domain structure of the aphid RlpA protein and structures of the corresponding mRNA and genomic DNA. For reference, the domain structure of *E. coli *RlpA is also shown. (C) Alignment of ICK motifs of the aphid RlpA with those of three antimicrobial peptides of the harlequin beetle, *Acrocinus longimanus*. Asterisks indicate the residues conserved in all the sequences. The grey background indicates conserved cysteines. The percentage of identity and *E*-value of bl2seq performed between each sequence and the ICK motif-1 (the one on the N- terminal side) of the pea aphid RlpA are shown on the right. Dashes (-) indicate alignment gaps. Dots (.) represent residues identical to those of the pea aphid RlpA.

In the previous study, the sequences of the transcripts corresponding to the cDNA clusters (unigenes) R2C00193 and R2C00214 were not fully determined, as the cap-trapper cDNA clones were sequenced only from the 5' end. In the present study, all the cap-trapper clone inserts relevant to these unigenes were amplified by PCR using vector primers (T3 and T7) and sequenced from both ends to obtain full-length sequences. In the case of R2C00214, all of the four clones had an identical sequence of 1312 bp encoding a polypeptide of 226 amino acid residues (Figure 1). Full-length sequences for R2C00193 were approximately 1 kb in length, with slight variations mainly in the putative untranslated regions (UTRs). They encoded a polypeptide of 220 amino acid residues (Figure 2). These full-length unigenes are hereafter referred to as R2C00214F (DDBJ: AB435382) and R2C00193F (DDBJ: AB435384 and AB435385), respectively.

### Putative LD-carboxypeptidase

Basic Local Alignment Search Tool (BLAST) search demonstrated that the product of R2C00214F has significant sequence similarity to the bacterial enzyme LD-carboxypeptidase (LdcA), and the microcin C7 self-immunity protein (MccF) that are produced by Gram-negative bacteria (Figure 1A). The top BLAST hit for the R2C00214F product was the hypothetical protein WD1015 [*Wolbachia *endosymbiont of *Drosophila melanogaster *(*Wolbachia *wMel; α-Proteobacteria, Rickettsiales)] (RefSeq: NP_966741) (*E *= 1 × 10^-23^), which has not been fully annotated; however, the analysis of the conserved domains of the gene product performed using the CD-search at the National Center for Biotechnology Information (NCBI) website indicated that the gene encodes the bacterial LdcA belonging to the peptidase S66 family (pfam02016, *E *= 1 × 10^-47^). The subordinate hits were either LdcA or MccF, the latter of which mediates resistance against microcin C7, an antimicrobial peptide that is secreted by enterobacteria and inhibits the growth of bacterial species phylogenetically related to the producing strains [35]. The mechanism through which MccF mediates resistance against microcin C7 is uncertain; however, MccF belongs to the peptidase S66 family, and all the residues required for LdcA activity are conserved in it [36]. Thus, in this paper, we collectively refer to these proteins belonging to the S66 family as 'LdcAs'. Putative orthologs of R2C00214F are found in a variety of bacteria, but not in eukaryotes, except for the fungus *Gibberella zeae *(RefSeq: XP_383840), implying that the two distantly related organisms, namely, the aphid and the plant pathogenic fungi, independently acquired *ldcA *from a bacterium via LGT. We discuss this possible mode of inheritance via LGT below.

R2C00214F appeared to lack the sequences required to encode the middle region of canonical LdcAs (Figure 1). To check the corresponding genomic sequences, the preliminary genome assembly of the pea aphid (Acyr_1.0) was screened using R2C00214F as the query sequence. The entire coding sequence (CDS) of *ldcA *was located in a single scaffold; however, the genomic DNA had a sequence corresponding to the middle region of the LdcAs that was missing from the R2C00214F gene product. This suggests that the sequence fragment represents an intron of the R2C00214F gene (Figure 1). In order to search for splice variants, we further amplified cDNAs for the aphid LdcA by RT-PCR using specific primers and determined their sequences. Unexpectedly, the amplified cDNAs essentially consisted of a single type of sequence variant that contained a sequence (402 bp = 134 amino acids) corresponding to the middle region of LdcA. This long form of the transcript (DDBJ: AB435383) encoded a 360-amino acid-long polypeptide sequence, while the short form (R2C00214F, DDBJ: AB435382) encoded a 226-amino acid-long polypeptide sequence (Figure 1). The long form and the short form appeared to be splice variants as cap-trapper libraries rarely contain inappropriate artifacts that do not reflect the mRNA structures *in vivo *[37,38]. The terminal dinucleotides of the insertion sequence were GT-CG, which is similar to the canonical splicing signal GT-AG. It has previously been verified that the GT-CG set can also be used as a splicing signal [39]. The short form of the transcript was not detected by RT-PCR; this might, at least in part, reflect the low level of expression of this truncated form of the transcript in the sample used in the study. The inconsistency might also be due to some bias in constructing the cDNA library in the previous study and/or in the RT-PCR employed in the present study.

Figure 1B shows the intron-exon boundaries of the aphid *ldcA *gene. The long-form transcript consists of two exons and a single intron. Only the second exon encodes the open reading frame (ORF) of the protein. The short-form transcript consists of three exons and two introns; the middle region of the second exon of the long-form transcript is spliced out as the second intron. These exon/intron organizations were verified by PCR cloning.

The long-form transcript was also characterized by BLAST similarity search. Once again, the top BLAST hit was the hypothetical protein WD1015 (*Wolbachia *wMel) (RefSeq: NP_966741) (*E *= 5 × 10^-81^). The subordinate hits were similar to those obtained with the short-form transcripts, but with much smaller *E*-values. The amino acid sequence of the long-form transcript exhibited 45% and 24% identity to the LdcA proteins of *Wolbachia *wMel and *Escherichia coli*, respectively (Figure 1A). Three catalytically active sites identified in *Pseudomonas *LdcA (Ser-126, His-304, and Glu-227) [36] were conserved in the aphid LdcA (Figure 1A). No other domain structure was observed in the protein.

### Putative rare lipoprotein A

The BLAST search revealed that the R2C00193F gene product is significantly similar to a bacterial protein, rare lipoprotein A (RlpA) (Figure 2A, 2B). The top BLAST hit was a putative RlpA family protein [*Bradyrhizobium *sp. BTAi1 (α-Proteobacteria, Rhizobiales)] (RefSeq: YP_001239851) (*E *= 9 × 10^-15^), and essentially all of the subordinate hits were thus annotated rare lipoprotein A. Homologous sequences of the pea aphid putative *rlpA *gene were observed in various bacteria, but not in eukaryotes, except for two other aphid species, *Aphis gossypii *(GenBank: DR391796) and *Toxoptera citricida *(GenBank: CD450666). Domain analysis revealed that the region detected by the similarity search corresponds to the double-ψ β-barrel (DPBB) fold, which is the domain conserved in RlpA proteins. Although the function of RlpA is not well understood [40], the DPBB fold is suspected to be an enzymatic domain [41].

Using RT-PCR cloning, two types of sequences were identified. As expected, these sequences corresponded to the transcripts originally found in the sequence cluster of R2C00193F (DDBJ: AB435384 and AB435385). These contained putative full CDSs encoding 220-amino acid polypeptide sequences (Figure 2A, 2B). These sequences appeared to be from distinct alleles, with two nucleotide discrepancies in their CDSs resulting in a single amino acid difference (denoted with an asterisk in Figure 2A).

Three other domain structures were observed in the pea aphid putative RlpA (Figure 2A, 2B). At the N-terminal region, a eukaryotic signal peptide motif was identified. BLAST search of the remaining sequences revealed that two regions adjacent to the DPBB domain are similar to the inhibitor cysteine-knot (ICK) motif of three antimicrobial peptides – Alo-1, Alo-2, and Alo-3 – of the harlequin beetle *Acrocinus longimanus *(Swiss-Prot: P83651, P83652, P83653) [42] (Figure 2C). The ICK motif presents a unique knotted topology of three disulphide bridges, with one disulphide penetrating through a macrocycle formed by the other two disulphides and interconnecting the peptide backbones [43]. The ICK family proteins are relatively small (typically less than 40 residues in length), and are found in various lineages of eukaryotes including plants, molluscs, arachnids and insects, exhibiting various biological activities such as toxic, antimicrobial, and insecticidal activities [42,43]. This motif was observed also in the putative ORFs of two other aphid transcripts. However, the domain has never been found in bacterial proteins, including RlpA.

To reveal the exon/intron structure of the pea aphid putative *rlpA*, a preliminary genome assembly of the pea aphid (Acyr_1.0) was screened using R2C00193F as the query sequence. The pea aphid *rlpA *locus was split into two distinct scaffolds (Figure 2B). The *rlpA *gene consists of three exons and two introns. The first exon contains the eukaryotic signal peptide, the second contains one of the cysteine-rich domains, and the third contains the DPBB domain and another cysteine-rich domain. The boundaries of the protein domains were consistent with the locations of introns. The chimeric structure of the aphid RlpAs might have come into being as the result of exon-shuffling [44] involving prokaryotic and eukaryotic elements.

### Pea aphid ancestor acquired *ldcA *via LGT from a *Wolbachia*-like bacterium

**Figure 3:**
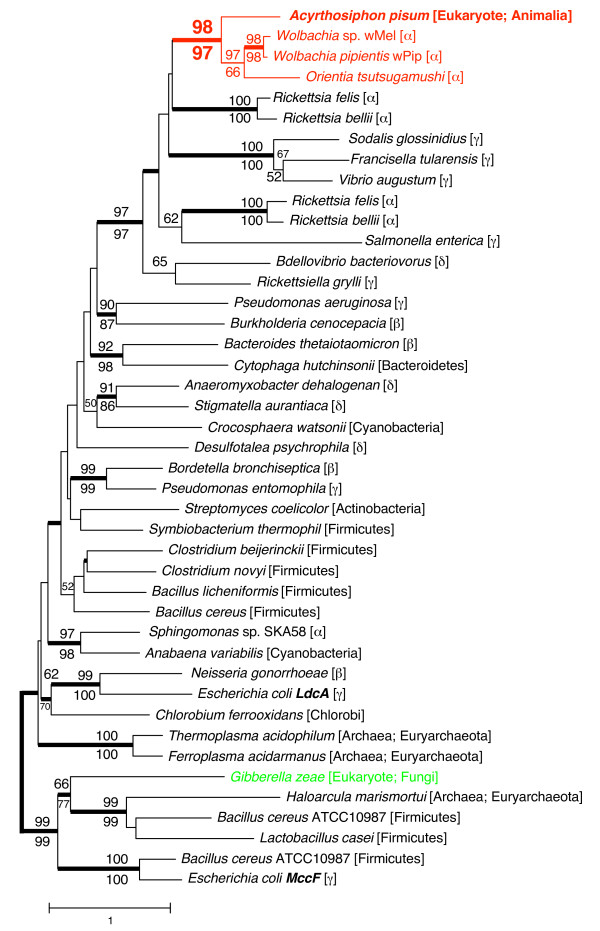
**Phylogenetic position of the aphid LdcA**. A total of 246 aligned amino acid sites were subjected to the analysis. A neighbour-joining tree is shown, while the ML tree and BP tree exhibited substantially the same topologies. On each node, bootstrap support values over 50% are shown (NJ above, ML below). Thickened nodes indicate the Bayesian posterior probabilities are > 0.95. Taxonomic positions (eubacterial taxonomy unless otherwise stated) are shown in brackets. α, β, γ, and δ indicate proteobacterial classes. The *A. pisum*-*Rickettsiales *cluster is shown in red. The sequence from the fungus *G. zeae *is shown in green.

The amino acid sequence of the aphid putative LdcA was subjected to molecular phylogenetic analysis (Figure 3). The phylogenetic tree demonstrated with robust statistical support (98% in NJ; 97% in ML; 1.0 in Bayesian) that the aphid gene is most closely related to the clade of LdcAs of rickettsial bacteria, especially *Wolbachia *(RefSeq: NP_966741) and *Orientia tsutsugamushi *(RefSeq: YP_001248242). This branching pattern can be most simply explained by the hypothesis that the aphid acquired *ldcA *via LGT from *Wolbachia *or some other rickettsial bacteria, many of which are known to be intracellular symbionts of insects. The putative orthologous gene detected in the plant pathogenic fungus *G. zeae *was distantly related to the aphid *ldcA *(Figure 3); this further suggests that the ancestors of *A. pisum *and *G. zeae *independently acquired the genes from different lineages of bacteria.

### Common ancestor of three species of aphids acquired *rlpA *via LGT

**Figure 4:**
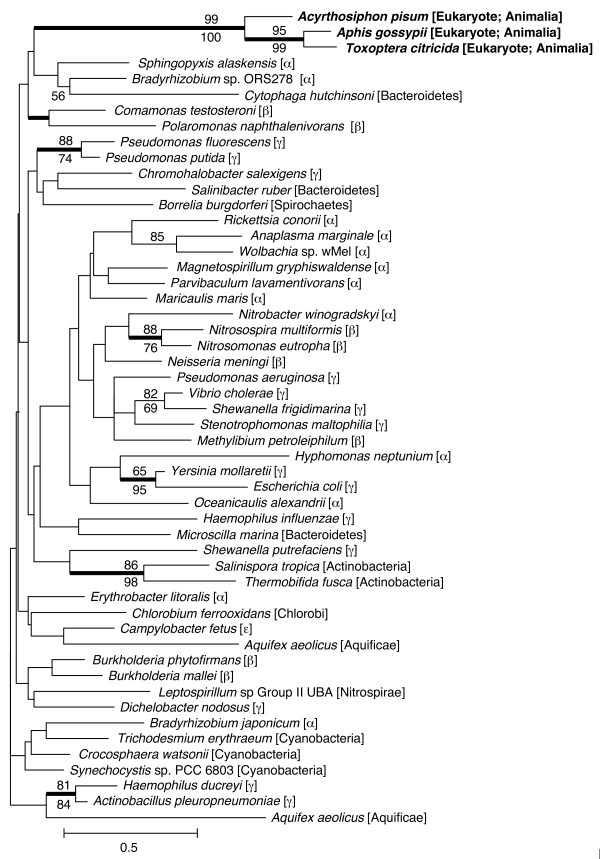
**Phylogenetic position of aphid RlpA**. A total of 76 aligned amino acid sites were subjected to the analysis. A neighbour-joining tree is shown, while the ML tree and BP tree exhibited substantially the same topologies. On each node, bootstrap support values over 50% are shown (NJ above, ML below). Thickened nodes indicate the Bayesian posterior probabilities are > 0.95. Taxonomic positions (eubacterial taxonomy unless otherwise stated) are shown in brackets. α, β, γ, and ε indicate proteobacterial classes.

The amino acid sequences of putative RlpAs of *A. pisum*, *A. gossypii*, and *T. citricida *were subjected to molecular phylogenetic analysis with RlpAs of various bacterial lineages (Figure 4). The highly conserved DPBB domains were aligned and used for this analysis. The phylogenetic positions of aphid RlpAs were not clearly resolved with a high level of statistical support. However, to date, no *rlpA *genes have been observed in any eukaryotes, except aphids. Moreover, the phylogenetic tree showed that the aphid *rlpA*s are monophyletic and that the phylogenetic relationships were congruent with the species tree of aphids [45]. This suggests that the common ancestor of these three aphid species acquired the *rlpA *gene from a bacterium via LGT. The relatively low resolution of the phylogenetic positions of the aphid *rlpA *may be partly due to the high evolutionary rate of the aphid lineages (see below).

### Aphid *rlpA *encodes a functional protein

To test the integrity of the functionality of the aphid RlpAs, *K*_*A*_/*K*_*S *_ratios of the DPBB-encoding sequences of aphid *rlpAs *were calculated. The ratios between *A. pisum *and *A. gossypii*, and between *A. pisum *and *T. citricida *were 0.45 and 0.30, respectively. Both of the ratios were significantly smaller than 1 (*p*-values were 0.02 and 0.001, respectively). This indicates that the aphid genes are not pseudogenes, but are functional and contribute to the fitness of the insects. However, the *K*_*A*_/*K*_*S *_values were somewhat higher than those of other bacteria [for example, 0.03, *Nitrosomonas eutropha *(RefSeq: NC_008344) vs. *Nitrosomonas europaea *(RefSeq: NC_004757); 0.04, *Yersinia mollaretii *(RefSeq: NZ_AALD01000017) vs. *Yersinia bercovieri *(RefSeq: NZ_AALC01000036); the *K*_*S *_values between other pairs were saturated]. This indicates that the selective constraints on amino acid substitutions in the DPBB domains are 8 to 15 times more relaxed in aphids than bacteria, under the assumption that synonymous sites evolve neutrally.

### *ldcA *and *rlpA *are highly expressed specifically in the bacteriocyte

**Figure 5:**
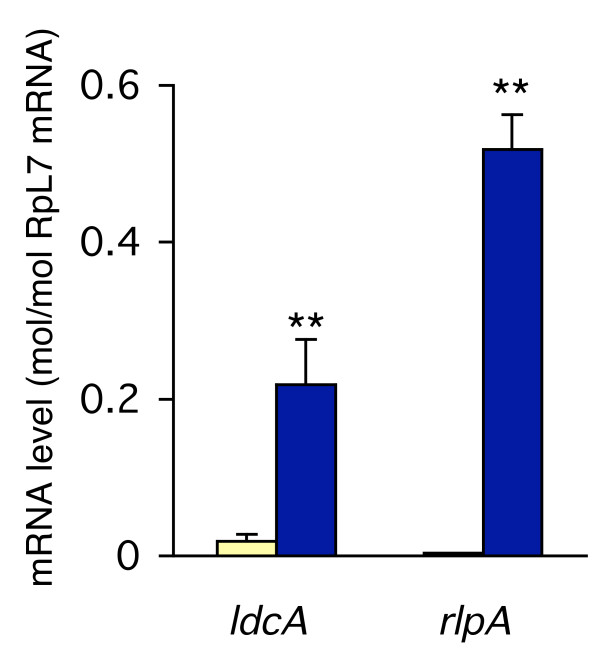
**Expression levels of *ldcA *and *rlpA *in the bacteriocyte**. Ivory columns, expression levels in the whole body; blue columns, expression levels in the bacteriocyte; bars, standard errors (*n *= 6). The expression levels are shown in terms of mRNA copies of target genes per copy of mRNA for RpL7. Asterisks indicate statistically significant differences (Mann-Whitney *U*-test; **, *p *< 0.01).

To examine the expression profiles of *ldcA *and *rlpA*, we quantified their transcripts in the bacteriocyte and in the whole body using real-time quantitative RT-PCR (Figure 5). The results clearly demonstrated that *ldcA *and *rlpA *are actively transcribed in the bacteriocyte. Transcripts for *ldcA *and *rlpA *were 11.6 and 154-fold more abundant in the bacteriocyte than in the whole body, respectively. It is also notable that the copy numbers of their transcripts in the bacteriocyte were comparable to those of the control transcript encoding ribosomal protein L7 (RpL7), indicating that their expression levels are relatively high. High levels of expression of these genes in the bacteriocyte strongly suggest that they are not only functional, but they play important roles in maintaining the symbiotic relationship with the obligate mutualist, *Buchnera*.

## Discussion

### Aphids have recruited genes from bacterial genomes via LGT

We have obtained strong evidence for two cases of bacteria-to-insect LGT. The genes encoded in the aphid genome that are expressed in the bacteriocyte were demonstrated to be significantly similar only to the bacterial genes, *ldcA *and *rlpA*. Quantitative RT-PCR further verified that these genes are highly expressed in the bacteriocyte. The orthologs of such genes are absent in *Buchnera*, the obligate mutualistic bacteria that are harboured in the bacteriocytes. These findings imply that the aphid *ldcA *and *rlpA *have compensational functions to support the survival of *Buchnera*.

Although until recently it was believed that LGT plays an important role exclusively in the evolution of unicellular organisms, especially prokaryotes [46-48], the accumulating genomic data is now revealing that LGT also affects the genomic content of multicellular eukaryotes with segregated germ cells. DNA sequences with significant similarity to genes of *Wolbachia*, an endocellular rickettsial bacterium, have been observed in the genomes of a wide range of arthropods and filarial nematodes [49-52]. *Wolbachia *is a maternally transmitted endosymbiont that can enter the germ line of host animals [34], which facilitates bacterial DNA transfer to the host nucleus. However, many of the transferred *Wolbachia *genes appear to be in the process of pseudogenization, and even intact "genes" are not expressed at a significant level [51,52], implying that these transferred genes do not confer novel functions on the host organisms.

The cases discovered in the present study are especially interesting in that these transferred bacterial genes not only retain their functionality, but are highly expressed in the bacteriocyte, which is the cell that harbours *Buchnera*. The molecular phylogenetic analysis clearly indicated that the aphid *ldcA *is closely related to that of *Wolbachia*, and of other rickettsial bacteria. Although infections of *Wolbachia *and *Rickettsia *are sporadically observed among the aphid species [17,23,25], the ISO strain that was used in the present study lacks such symbionts [33]; this suggests that the previous infection left only a transferred gene as a footprint, while the source bacterium disappeared. With regard to *rlpA*, it was clearly demonstrated that this gene also was of bacterial origin, but its phylogenetic position has not been fully resolved.

### Eukaryote-type structures of the genes and transcripts

Recent studies have revealed that LGTs from bacteria can occur in metazoa [49-52]. However, these transferred genes cannot function unless they obtain eukaryotic promoters, since the gene expression systems of prokaryotes and eukaryotes differ. The likelihood of promoter acquisition seems very low, as suggested by the previously reported lack of expression of laterally transferred genes [51,52]. The aphid *ldcA *and *rlpA *are highly expressed in the bacteriocytes, clearly indicating that these genes have acquired eukaryotic promoters, although the mechanism of promoter acquisition has yet to be determined. The cDNAs for the aphid *ldcA *and *rlpA *were originally observed in a cDNA library constructed by the cap-trapper method that targets the 5' cap structure and 3' poly-A tails of eukaryotic mRNAs [37,38]. This suggests that both the mRNAs for the aphid *ldcA *and *rlpA *have the 5' cap structure and 3' poly-A tail that bacterial mRNAs lack. Indeed, polyadenylation signals (AAUAAA) are observed in the 3'-UTR of the transcripts. Screening of the genome scaffold followed by PCR cloning revealed that the genes have spliceosomal-type introns. This type of intron has not been observed in bacterial genes, suggesting that these genes acquired introns after they were transferred into the aphid genome.

### LdcA may be used to control *Buchnera*

LdcA is an enzyme required for recycling murein (peptidoglycan), a component of the bacterial cell wall. LdcA releases the terminal D-alanine from L-alanyl-D-glutamyl-*meso*-diaminopimelyl-D-alanine, which contains turnover products of murein. The disruption of *E. coli ldcA *results in bacteriolysis during the stationary phase, indicating that the reaction is essential for bacterial survival [53]. In the mutant, due to a defect in murein recycling, the unusual murein precursor uridine 5'-pyrophosphoryl *N*-acetylmuramyl-tetrapeptide accumulates, and the overall cross-linkage of murein decreases dramatically. This is interpreted as a reflection of the increased incorporation of tetrapeptide precursors that can only function as acceptors and not as donors in the cross-linking reaction.

*Buchnera *has cell walls composed of murein [54], but it lacks *ldcA *[10]. Although the evolutionary origin of the aphid *ldcA *seems to be from rickettsial bacteria and not from *Buchnera*, it is intriguing to note that this gene is highly expressed in the bacteriocyte. Aphids may control the proliferation of *Buchnera *using *ldcA*, which was recruited from another symbiotic bacterium that previously had resided in aphids.

### Chimeric structure of putative RlpA

The molecular phylogenetic tree indicated that the LGT of *rlpA*s occurred before the divergence of the three aphid species. On the basis of fossil records, this divergence is inferred to date back to more than 50 million years ago [45,55]. Even if the transferred genes successfully acquire sequence elements that allow their expression, contribution of the genes to the host fitness, or strategies enabling the selfish propagation of the genes, would be required for the maintenance of the transferred genes in the host genome for such a long period of time. The functional role of the *rlpA *in any bacteria is not well understood; however, RlpA suppresses the *E. coli *mutant of Prc that cleaves the C terminus of FtsI [40], suggesting that *rlpA *plays an important role in bacteria. Domain analyses revealed that, in addition to the conserved DPBB domain, the aphid RlpA has three other domains that are not found among bacterial orthologs. This implies that RlpA might have gained novel functions that are yet to be determined. Although the function of RlpA is not well understood, the high level of expression of the aphid *rlpA *in the bacteriocyte implies that this gene is also essential for *Buchnera*.

## Conclusion

In this study, several lines of evidence indicated that aphids acquired genes from bacteria via LGT, and are using such genes to maintain the obligately mutualistic bacteria, *Buchnera*. Phylogenetic analysis clearly demonstrated that one of the genes was derived from a rickettsial bacterium that is closely related to the extant *Wolbachia*. This is the first report of functional genes that were laterally transferred from symbiotic bacteria to metazoa. The cases presented here are of special interest in that these transferred bacterial genes not only retain their functionality, but are highly expressed in the bacteriocyte that is differentiated so as to harbour *Buchnera*, which lack such genes.

## Methods

### Aphids

Strain ISO, a parthenogenetic clone of the pea aphid *Acyrthosiphon pisum *which is free from secondary symbionts including *Wolbachia*, was used in this study. The insects were reared on *Vicia faba *at 15°C in a long-day regime of 16 hr light and 8 hr dark. Parthenogenetic apterous adults (12 to 15 days old) were used for the experiments.

### Cloning of the pea aphid genes

Genomic DNA was extracted from the whole body of the pea aphid using a DNeasy Kit (Qiagen). Total RNA was extracted from bacteriocytes and first strand cDNA was prepared as described previously [33]. PCR was performed using various sets of gene specific primers (Additional file 1). PCR products were either purified and sequenced directly or cloned using the pGEM-T easy vector system (Promega).

### Characterization of gene products by similarity searches

Homologous protein sequences and conserved domains were detected by BLASTP similarity searches at the website of the NCBI using deduced amino acid sequences as queries [56]. The presence and location of signal peptides were predicted using the program SignalP 3.0 [57]. Statistical tests of homology between two amino acid sequences were conducted with bl2seq. Default parameters were used except for the matrix (-M), set to BLOSUM80, the gap existence penalties (-G), set to 11, and the theoretical database size (-d), set to 127,836,513, the size of Swiss-Prot release 55.0.

### Molecular phylogenetic analysis

Multiple protein sequences were aligned using the program package MAFFT 5.8 [58], followed by manual refinement. Amino acid sites corresponding to alignment gap(s) were omitted from the data set. Only unambiguously aligned amino acid sequences were used for the phylogenetic analysis. The aligned sequence data are shown in Additional file 2.

Phylogenetic trees were inferred by the neighbour joining, the maximum likelihood and the Bayesian methods. Neighbour-joining trees were constructed using the program package Xced [59]. The distance matrix was estimated by the maximum likelihood distance method assuming the JTT model with among-site rate heterogeneity. The bootstrap probability for each node was calculated by generating 1000 bootstrap replicates. Maximum likelihood trees were estimated using the program package RAxML [60]. In the analysis, the JTT model was used as a substitution model for amino acids. To incorporate the effect of among-site rate heterogeneity, a mixed model (one invariable rate plus Γ distributed rates) was used. The support values for the internal nodes were inferred by 1000 bootstrap replicates. In the Bayesian inference, we used the program MrBayes 3.1.2 [61]. The JTT +Γ +Inv model was used as a substitution model. In total, 4100 trees were obtained (ngen 410,000, samplefreq 100), and the first 2000 of these were considered as the 'burn in' and discarded. We checked that the potential scale reduction factor was approximately 1.00 for all parameters and that the average standard deviation of split frequencies converged towards zero. *K*_*S *_and *K*_*A *_values were calculated as described previously [62]. Statistical significance of the obtained *K*_*A*_/*K*_*S *_value was tested against a bootstrap distribution of *K*_*A*_/*K*_*S *_values, which was generated by 10,000 bootstrap resamplings of codons from the original alignment.

### Real-time quantitative RT-PCR

RNA was isolated from whole bodies and bacteriocytes of 12 to 15-day-old parthenogenetic apterous adults using TRIzol reagent, followed by RNase-free DNase I treatment. Each whole body sample and bacteriocyte sample was derived from one individual and a batch of bacteriocytes that were collected from about 10 individuals, respectively. First-strand cDNAs were synthesized using pd(N)6 primer and PrimeScript reverse transcriptase (Takara). Quantification was performed with the LightCycler instrument and FastStart DNA Master^PLUS ^SYBR Green I kit (Roche), as described previously [33]. The primers used were: ldcA-677F (CAACCTGACGCTAGTCGAGAACT), ldcA-758R (CACGTCCTCCAAGAACACGAT), rlpA-449F (CGGCGGACGGTAAGGTAAT), and rlpA-529R (ACTGTACCGGGCCTGTGTTC). The running parameters were: 95°C for 10 min, followed by 45 cycles of 95°C for 10 s, 55°C for 5 s, and 72°C for 4 s. Results were analyzed using the LightCycler software version 3.5 (Roche), and relative expression levels were normalized to mRNA for the ribosomal protein RpL7. Statistical analyses were performed using the Mann-Whitney *U*-test.

### Source of the genomic data of the pea aphid

The preliminary genome assembly of *A. pisum *was obtained from the Human Genome Sequencing Center at Baylor College of Medicine through the web site at .

## Authors' contributions

AN conceived the study, performed the molecular work, and contributed to the structural analysis. NN performed the structural and phylogenetic analyses. NN and AN collaboratively designed the study and prepared the manuscript. Both authors read and approved the final manuscript.

## Supplementary Material

Additional file 1 **Table S1**. Primers used for cloning the aphid *ldcA *and *rlpA* Click here for file

Additional file 2 **Alignments of the amino acid sequences of (A) LD- carboxypeptidase (the product of *ldcA*) and (B) rare lipoprotein A (the product of *rlpA*)**. Asterisks (*) and circles (O) represent amino acid positions that are occupied by identical and chemically similar amino acids for all the sequences compared, respectively. Gaps (-) were inserted to increase sequence similarity. Dots (.) represent residues identical to those of the pea aphid proteins. Ambiguously aligned regions are trimmed and the numbers of the amino acid residues trimmed are shown in parenthesis. The GenBank accession numbers of sequences used are included in the additional file itself. Click here for file
