# Supplementary material for: Aphids acquired symbiotic genes via lateral gene transfer
Source: BMC Biol. 2009 Mar 10;7:12. doi: 10.1186/1741-7007-7-12 (PMC2662799; doi:10.1186/1741-7007-7-12)
Supplement: Additional file 2 — Alignments of the amino acid sequences of (A) LD- carboxypeptidase (the product of ldcA) and (B) rare lipoprotein A (the product of rlpA). Asterisks (*) and circles (O) represent amino acid positions that are occupied by identical and chemically similar amino acids for all the sequences compared, respectively. Gaps (-) were inserted to increase sequence similarity. Dots (.) represent residues identical to those of the pea aphid proteins. Ambiguously aligned regions are trimmed and the numbers of the amino acid residues trimmed are shown in parenthesis. The GenBank accession numbers of sequences used are included in the additional file itself. [file 1741-7007-7-12-S2.pdf]

A

```
Acyrthosiphon pisum          (22)VRVIAPSSMGKPSN-----LEVFRRFLRDA-GMTARVDDSIYRA--GADPFYANTDQFRAADLVDA--LTD-SCTVVWCARGGKGASRMIPYLEALPADRKQRIRDA--RKTLVGY
Wolbachia sp. wMel           (23).DI.....K..E.D-----.TTIKEYVEAL-DFNPHISEK..SN---DN...S.S.E...N...S.--..G-DSKII..I...E....L.....K..N.K.E..AQN--K.I.I..
Wolbachia pipientis wPip     (19).DI.....K..E.D-----.PTI.EYVKTL-.FNPHISEK..SN---DN...S.S.E...N...N.--...-DSKII..I...E....L.....K..N.K.E..AQN--K.I.I..
Orientia tsutsugamushi       (25)ID......K.SEL.-----.QLIEDY.ESI-.VK.N.PEN..SD---NE.L.S...E...ES..K.--..NPKSIAI..I...R....L.....K..D.V.KK.AQNKN..I.M.F
Rickettsia felis             (29)IT.V..ATGADNKT-----.SDLKN-INGL-NLQILSKCFAKGK----L..L.SS.EV.FNC.R..--.F.E.DN...SL...Y.SA.I..G.LK.SKPN.--------E.FFI..
Rickettsia bellii            (32)IN.V..A.GADNKT-----.SDLKN-IKTL-NLNMPTKCLSKGD----L..L.S..EI.FNC.K..--.YNE.SNI..SL...Y.SA.L..D.LK.SKPN.--------E.FFI..
Sodalis glossinidius         (47)IYL.SS..QYDENT-----IGEIKAVFQQQ-.YSVDSRYLDQQP---TPLG.V...EV..ET.IN.--...ENVKYL.FV...S..LNLY...YRNRDKIAAAS-----P.I.I.F
Francisella tularensis       (33).AL.NV.TQYY.ND-----IKQAEKA.K.T-.YNTTYKYLDIYP---S.FG.S.P.SI..KI.L..--.L.KNIDII.FLK..G..FNLL...YDHINEL.KAK-----P.I...F
Vibrio augustum              (21)IAL.SC.TQYDEQS-----VNNVVDTFIAQ-.YMVTTKYLNQVI---S.IG.V....E..NN.IA.--.L.PTID.L.FF...G..LNLL.F.H.YKTQLLEIK-----P.MI..F
Rickettsia felis             (12)ISI...A.GCLDAR---DK.KEAIKI.ALH-.FKTLI..K.FLG--DELL.F.APKEE.LRMFKE.--MENEQVKII.AF...Y.C.EFVEDCFNIKQKG---------D.I.I..
Rickettsia bellii            (12)ISI...A.GCPDAQ---DR.AKSIEM.NLQ-.FKTL...K.LVG--DEL..FVAPKAE.LRMFKE.--MENEQVKII.AF...Y.C.EIVEDCFNIQKKG---------D.I.I..
Salmonella enterica          (12)ID......PIDNPQ---ERYKKIEQYFKENTPFKINIP.HLIEP--T.LLDE...IWK..KFVY..--FCS-ESKAI.AIS..GWGASILGE.MSY.KPD.--------I.PII..
Bdellovibrio bacteriovorus   (11)ID.V..GYPS..EE-----V.GA.A..EKW-NLQP.IPKG.IKP----HFLH.HE.EA.WQFMKA.--IESKDSR.I..L...Y.SN.L..F.AKMKKPKE--------P.L.I.I
Rickettsiella grylli         (20).DI...A.GVSDTS----TMRKLKNL.ESW-QLIP.ISS.LLGS----.LLC..S.EN.FQH.QE.--.SNTDSQAI..L...Y.CT.L....LK.T.PE.--------C.LFI.F
Pseudomonas aeruginosa       (17).AL...A.AIATDV-----..ATL.Q.EVH-.VDYHLGRHVEAR----YRYL.G.VEQ.LE..HN.--FDMPDI.A...L...Y.CGQLL.G.DWGRLQAAS-------PRP.I.F
Burkholderia cenocepacia     ( 7).A.V..AGIPDRD.-----.ARAVSL.ESW-.FSVS.GAHVTDR----FRYL.GSR.D.ID..LA.--.S.PAVDI..I....Y.STHVL.A.PTTVPR----------K..VI.F
Bacteroides thetaiotaomicron (14).VIVS...KIDKEF-----.KRAKKR.ESW-.LKVS.GKYAGGS----SGR..G.VRQ.LQ..QS.--MD.PKVKAIL.S...Y..VHLVDKIDFTAFREH--------P.W.L.F
Cytophaga hutchinsonii       ( 6)IH.VS.AGAVNKI.-----.AAGISYVSGF-DFLIKAGK..FQK----KGYF.G..TD.QY..QL.--.D.PNALAI.V....Y.TT.I.DTISWKKFFK.--------PSWII.F
Anaeromyxobacter dehalogenan (12)........PFE.EQ-----.ARGLAV...RL.LEP.MRADLGAR----ARYL.GD.AR.LEEWRE.--VA.PDARAIF.....F....LL.EVDPA.LLER--------P.A...F
Stigmatella aurantiaca       (31).H.V..AGPFDKAG-----F.AGLAIIGQ--RYSPVYGPDLFSS----HRYL.GQ.SR.REE.SRV--.R.PQGRAIFA....Y.SM.LL.G.PLAE.G----------PSA...F
Crocosphaera watsonii        (18)IVG.S..GPI.DVT----AI.KAQEIM.SQ-.YNLELGKHWNDQ----YGYL.G..EQ.RD..LE.--FKNPNYKAIIAV...Y..A.LLEEKDWKTIFTNY-------P.W.I.F
Desulfotalea psychrophila    (24)IG.....GRLSN.D----AFYKGLHI.EGF-.YRL.FPRKLWPG----SDYL.D..EN..LEFHKM--WA.EEVQALI.M...Y.VL..LEF..LKEIKKT--------P.Y.L.F
Bordetella bronchiseptica    ( 9)IAIV..A.AIAGAA------DEAAEW.LER-.YEPQ.MPAARARLAPPFDYL.GD.AA.L...HA.--FA.PAVGA...LQ..F.SW.VLDQIDYGLLR.H--------A.PFI..
Pseudomonas entomophila      (20)FAIV..AGPARVDA------DKA.QWFERR-.YRC.LYPGVSQ.----.GYL.GS..Q.LQ..H..--FR.PGIDAIL.M...Y.SM.LLDA.DLELIR.H--------P.P.I..
Streptomyces coelicolor      (15).A.V...GPVPEER-----.QAGLDV..GW-DLDPV.APHVLDR-HDTFDYL.G..AD.....QA.--WC.PAVDA.L.....Y.VQ..ADL.DWEAMRAAG-------P.VF..F
Symbiobacterium thermophil   (17).G.V..AGPVP.EA-----..EGL.R..AW-.YRTV.GEAVLDR----RGYL.GA.DR..E.FNRM--WA.PAVDA.I.....Y..M.ILRRIDWELIR.N--------P.FFC.F
Clostridium beijerinckii     (12)IGL..A..PT..DR-----I.PSIKAMEEL-.LEVVLGK.CKCY----HGYLSGS.EI..N.INKM--FD.KTIKGIFAI...Y..G.LLSM.DYNMIK.N--------P.VFA..
Clostridium novyi            (11)IGLVS...PEN.DK-----IKESIA..KEQ-.FKIKEGKHL.DK----RGYL.G..KN..E.FMNM--FL.DEVSMIL.V...Y..M.IL...DYEKIKEN--------P.I...F
Bacillus licheniformis       (13).G....A.PPV.EK-----.DRAV...KEL-.LEVK.GR.AAER----HGYL.GS.EE.LG..H.M--FE.R.VKA.I..C..Y.TA.IAGKIDYRLIKSR--------P.IFW..
Bacillus cereus              (12).MI....GPPTIE.-----VLKGVNV.QEM-.LSVIIGK.V.EK----YG.L.GS..V.LD.IHE.--F.NNEVKAIF.....Y.SA.LL.HIQYEVIRQN--------P.IFW..
Sphingomonas sp. SKA58       (45).GL.E.AGFTDDAF----D.DLVEDTI.AM-.LKPKRAPHLIDR----FGYL.GK.AY..S.VNAM--FA.PAVSAIFAV...W.CA.IL.L.DWRKIRSN--------P.L.I.F
Anabaena variabilis          (68).GL...AGTFE.RY-----I..VQQH.TNL-.LK.K.GRH.LDR----YGYL.GR.AD..Q.VN.M--FV.D.VQAILAM...W.CN.IL.L.NYPLIRSH--------A.I.M..
Neisseria gonorrhoeae        (61)L..V.S.GFAEDT.----RVNTALTR.YN.-.F.VTNQQAGS.R----FQRF.G..AQ....FQEVASGRVATPK.LMGL...Y..A.IL.HIDFASLGARM.EH----GTLFF.F
Escherichia coli LdcA        ( 3)FHL....GYCIKQH----AALRGIQR.T..-.HQVNNVEV.A.R----CERF.G.ETE.LE..NSLA-RLTTPN.I.LAV...Y....LLADIDWQALVAR.QHD----PLLIC.H
Chlorobium ferrooxidans      (14)IGL.S...HCAYPV----KIGQAISY.EAN-.YRVKLSSHLN.I----.TDP.VA.REKLH.IHEM--FS.PDVRAIF.L...A.SA.LLNQ.DYTLIAAN--------P.I.A..
Thermoplasma acidophilum     (13)I.I...A.APDMK.-----.SRSIAR.KKS-.YRVTLGRN.KKL--MQMNQL.AP.TS.RDE.ME.--FL.DHVKAIF.....Y.SIHIL.LIDYDAIRDH--------P.IF...
Ferroplasma acidarmanus      (15)I.....A.APDMIK-----.SRGISK.KKL-.YKVT.GKN.KKL--NQRNDL.APAKD..EE.MS.--FR.DNVKAIF.....Y.SIHILSL.DYEAIREN--------P.IFM..
Gibberella zeae              (15)IAF.S..ERINSTL--PAVVDRASAV.SNK-.YHVQTFF.ED-------NGIQSCIDN.LSEIRA.--FS.P.IAA.ITTI..TTFTELL.A.I.DKELHEIIRAN---P.VV...
Haloarcula marismortui       (18).A.V..A.NAPE.A--RFIY.LGLERM.EVFDLDPVAYPTATAD----PEWL.DNPEA..EEIMK.--FR.PDISA.IANI..HDQIT.L.F.DGDVLREH--------PTRFY..
Bacillus cereus ATCC10987    (12)IGIYS...PVTYTS--PKRF.RAKSY.QQK-.FHILEGSLTG.Y----.FYRSGSI.E..EE.NVL--IRNPNISCIMSTI..MNSNSIL..IDYDAFLKN--------P.IMI..
Lactobacillus casei          (12).AIVSL.AGTLGESFAAHQ.QLGTER.KAM-KLEP.FMPNAL.G----QAYLKAHPEA.....KA.--FL.P.IKGII..I..DDTY.I....LDD..FTQAVADH---P.LFT.F
Bacillus cereus ATCC10987    (13).ATVS..WG.AGDSEIRWRY.QGVKR.EEVF.L.VVPMPNSLKG----SE.IY.NP.A..E..MT.--FQ.TRVKAIIANI..QDSI.LL..IDFNAIREN--------P.IFM..
Escherichia coli MccF        (18)IGFFSS.APATVTA--KNRFFRGVE..QRK-.FKLVSGKLTGKT----.FYRSG.IKE..QEFNEL--VYNPDI.CIMSTI..DNSNSLL.F.DYDAIIAN--------P.III..
Consensus                        O                                                   O                 O    **                               *
```

```
Acyrthosiphon pisum          SDATALHVYLQTVYGWQTVHGTMLEMIADG----------TVDRRSVDALVALVTGRSDRV(17)SLRSKVAGGNLTLVENSLGTS-CCFRG( 0)DGKIVFLE-------DVRVQPYAL
Wolbachia sp. wMel           ..I....IH..AK.D...L........VNN----------S.SES..EK.KE.ILNKR.SI(17)R.K..II...M......I..V-WQINA( 0)K...L..--------.I..Y...I
Wolbachia pipientis wPip     ..I....I...VK.D...L........VNN----------S.AES..EK.KE.ILNQHNSI(17)K.E..II...M......I..A-WQINA( 0)KN..L..--------.IK.Y..SI
Orientia tsutsugamushi       ..VSV..I...NK.D.S.L.SP...Q.V.N----------KISQD.I.K.EL.IS.YRNNI(16)I.E...V..SIS..RA.I..C-WQIDT( 0)KD..L..-------E.EPD.AL.
Rickettsia felis             ..I....LF.SQEW..K.I..SNIADLLKT----------DK.QSNFTK.AEILK.KVKQ.(16)LVNG.LT.....M.QT.I...-WQIKT( 0)K...L..--------..N.A.FR.
Rickettsia bellii            ..I....LF.SQEW..K.I...NVAGLLKP----------EQ.QGNFIK.AEILQ.KVKQA(16)LVSG.LT.....M.LS.I..R-WQIKT( 0)A...L..--------.IN.A..Q.
Sodalis glossinidius         ..V....HFINN.IK.PSI..ILASFNSEMYE--VDKTEQISMYSGIQQVFRTI.NGMTYT(11)GGQGTLN.......QSLFS.R-YEKNY( 0)AD.VMLM.-------.TG.TYKQ.
Francisella tularensis       ..V..I.FFVNN.L..KSL..VVAAYNKNAYSSQKIGKIRIN.LERIPNITEIINNGISYD(11)GIDGSIV...M..IYSYFS.V-YQQDI( 0)ST..L..--------.TGISFRQ.
Vibrio augustum              ..V..I.SFINNEL...S..AVNASTNQAT---------NGGNEQPIPD.KT.INDGVSYN(12).VSGQLI...H...SATF..H-YQPDF( 0)SN.VLL.--------.IG.TYRQ.
Rickettsia felis             ..I.V..LL.NNH.NIP.I..SV.TSLLPP------------TNQAIIPIIDVLE.KKSEI(11)NITG.IT.....VFSKLI...-IDLK-( 0)KDN.LL.--------..NEKA..V
Rickettsia bellii            ..I....LLFNGH.NIP.I..SV.TSFLHP------------VNQDINSILNVLK.EDS.I(11)PIEG.IL.....VFSKLI...-VNLK-( 0)TNN.LL.--------..NEKG..I
Salmonella enterica          ..I....I.ANEYLNFPSI.SVV.GING.I--------FPGYNKNGLG.TLDILS.KKTD.(15)EVTG.IV...SL..SALN.A.GLSLKT( 0)K..FL...-------SIADD.GQF
Bdellovibrio bacteriovorus   ..ISS..TFVNQEW..SSL.APL.DRLGRN----------L.SAKHEKEIHDILF.KTRQ.(16)T.K..IV.....VLQST...P-WQVDA( 0).RRLL.V.-------.IGERG.RI
Rickettsiella grylli         ..I....LF..QKWH...L..PS.NQVVHH----------FIETENSNE.KKIIF.QLSQL(15)..YAPLT..S.SI.QT..C.D-WHIET( 0)RD..L...--------.NESA.RI
Pseudomonas aeruginosa       ..ISV.LSAFHRH-.LPAI..PVATGLGLSPL--SAPREQQERLA.LASVSR.LA.IDHEL(10)RVEGALI.....ALACMA..LGGLHAP( 0)A.S.LV..--------.GEPY.R.
Burkholderia cenocepacia     ......FCA.KQIP.IDVI..PT.NGL.TK------------.EE.RLSVLGAL..V.PTP(11)PIGGPLV.....VLASMA.SR-WEP.F( 0)RDS..V..--------.TELA.RI
Bacteroides thetaiotaomicron ..I....NLF.KN-.YASL.SL.ARHLSVE----------PEEDPC.AY.KDILF.NLPVY(11)TAEGILR...MAVAYGLR..P-YDIPA( 0)E.T.L...--------.SER.H.I
Cytophaga hutchinsonii       ..I..F.IH.SNH-NIPSI..P.VAQA..D-----------NHKEA..LVT.ILKNDFPDY(11)KAKAPIT......ICSTI..A-TEIIT( 0).N..L.I.-------E.GEAA.RV
Anaeromyxobacter dehalogenan ..VC...AA.NRA-.LA....PV.TQLGRA------------PEAA.AQ.E..LM.GGP.P(21)RAAGPLL..S...LAHLC..P-FMP.L( 0)E.ALLL..--------.GEK..R.
Stigmatella aurantiaca       ..I....LA..AK-..VSL..PV.TQLGKQ------------PPEVHER.FQ.LESPAPAP(11)TAEGPLL....AVLTCLV..P-YLPSF( 0)E.AVLL..--------.GER..R.
Crocosphaera watsonii        ..I.S.LWK-RAKVKISSI..PV.TTLGQE------------PDW.LKR.FNYLK.DPLPP( 9)QGEGRLLPA...VAT.I...P-LQPPL( 0)NDV.LA..--------.GEA..K.
Desulfotalea psychrophila    ..ISL.QN..LDRI.LPAI..PVVTSL.TA------------S.S..R..Q.ML..DLQQM(13)GGTGTLV.....SLSTV...P-FDISW( 0)KD.F....--------.GEPI.RI
Bordetella bronchiseptica    ..V....LAI.RYA.FV.F..P..AQDLLA----------GRQAPTEQ..LDM.S..LGAG(17)VATGRLI....A.LAALT..R-YAIDA( 0)RDG.L.F.--------.NEALPRV
Pseudomonas entomophila      ..I....TAINRYS.LV.F..A..NADLL.----------DKLEPT.GS.FEQLG.QLGKG(17)VASGRLM....SMLGATM.SL-AEIDT( 0)RDS.L.I.--------.NEPL.RV
Streptomyces coelicolor      ..I....EAFA.RL.LV.L..P.AAG.DFI-----------KNA.AQEH.R.TLFAPETVR(12)RA.GVTL..C.A.LAAD...PHARPGA( 0)R.GLLC..--------.GEET.RI
Symbiobacterium thermophil   ..I....LAMAREA.LV.F..P.VAAFGGAHG--YNGAGLRRALQQ.GP.GEMPWPDPAEE(11)VAEGRLI......IVSL...P-WEPDF( 0)T.C..L..--------.DEA..RV
Clostridium beijerinckii     ..V....TVFNEKCKFI.F.TP.ISTEFYK----------G..DYTM.YFIKNIFSDKPLG(16)KAIG.LV....S..AS.M..P-YELDT( 0)K...L...-------E.DEY..KI
Clostridium novyi            ..I.V.LNSISSNCDFI.F..P.ATSNLKD------------KETYNSLFTT.M..TRPY-(16)IAEG.IV....S.IASTM..P-YEIDT( 0)KDN.L.I.-------.INEE..SI
Bacillus licheniformis       ..I.F..TAIRQNT.LI.F..P..SSDIGK---------DD.HPLTRRSFQQ.FQKMTLSY(13)AAEGELV....A.LVTT...P-YEIDT( 0)K..LL.I.--------.DEE..RI
Bacillus cereus              ..I....TAVSRYAKLI.F..P.I.ELGK.-----------I.SL.LSSFNQ.FHPY.TIL(12)TVTGTLV....AVLTSII.SP-YEINT( 0).NTLLL..-------.IGEE..RI
Sphingomonas sp. SKA58       ..I....LAFAAKA.FT.I..PNASSSWGQ--------------Y.W..FR.VAFDAGTPM(24)VA.GRLL.....VLAALM..P-WLPDF( 0)A.A.L.I.-------.IGEA..RI
Anabaena variabilis          ..I.S.LLAINARSRIL.F..PVATSTWTP--------------FMLEY.KRILFNAEAVT(17)KAQGQLV....SVLSAMV.SP-YLPGW( 0)QNR.L...--------.NEDV.RV
Neisseria gonorrhoeae        ..VC.VQLA.LAKGNMMSFA.P.AYSDFGK---------PAPGAFTM..FIKGA.QNRLT.(10)ETEGTLW....SVLASLA..P-YMPDI( 0)..G.L...--------.GE...RI
Escherichia coli LdcA        ..F..IQCG.LAHGNVI.FS.P..VANFGA---------DELNAFTEHHFWLALRNETFTI( 9)RAEGTLW....AMLISLI..P-WMPKI( 0)ENG.LV..-------.INEH.FRV
Chlorobium ferrooxidans      ..I...SLA.YAKT.LISFS.P.AATELYA-----------PTPYTEEHFWGML.SP.YAR(16).AEGQLI....SVLSAMI..P-FLPLF( 0)NDTLL...-------.INEPA.RI
Thermoplasma acidophilum     ..I....LA.NAKASLI.F..P.PAADP.K-----------FSKTDFKDFIDILK.ETTHL(14)KVEGLSM.T..SVFASLI..G-YLPDS( 0)Q...L.A.-------.TG.TSGDI
Ferroplasma acidarmanus      ..I....LAINRNSNLI.F..P.VASDV.D-----------ISKP.FKEFLGILS.K..EI(14)KTEGYSM.T.IS..ASL...N-YIPET( 0)TNR.F.I.-------.TD.TSGDI
Gibberella zeae              ..I.G..WF.YAMT.LR.FY.PGAVPEIGEPN--DINDKN.PLSFC..N.LRAIASPKPLG(39)KAQGRLF..C..V.ARLG.IRAIIPDW( 0)R.R.....TATNEDGSGGNP.HRV
Haloarcula marismortui       ..N.N.ALF.WNHGIVSYYG.ST.LEY.MD---------GEMFDYTKEY.RRALFED.VGE(39)TVSGRIW..CYAVLVEQFLADRYLPDP( 3)N.TVLA..TS-----ELIPD.AVV
Bacillus cereus ATCC10987    ......LLAIYAKT.IP.FY.PA.VPSFGE--------FEPFVDCTYKYFAETLLHDHSLP(36)KATGRII....NTIQGI..SP-YMPHI( 1)E.D.L.I.-------.SSKDAATI
Lactobacillus casei          ..T.ID.L-MFYQL.MTSFY.PNFLNDLAE-------LDTRMLPYTAASFQHYFKNPATTA(39)RVTGTLL..C.DSLHDLIYPVRYQDEP(13)TN..L.I.TS-----EDKIT.A.Y
Bacillus cereus ATCC10987    ..V.IS.L-FCHKA.LSSFY.PAILTDFAE--------NVEM.PYT.EMVNRTLFSNEMIG(39)TVQGRLI..CIEVL.FAK..E-LWPEK( 3)EDS.L.F.TS-----EDHPE.SYI
Escherichia coli MccF        ..T...LAGIYAKT.LI.FY.PA.IPSFGE--------HPPLVDITYESFIKIL.RKQSGI(38)KVEGR.I....NTLTGIW.SE-WMPEI( 1)N.D.L.I.-------.S.KSIATI
Consensus                    **                  O                                               O   OO                             *
```

Continued.

```
Acyrthosiphon pisum           ERSLDHMKLAGVFDGATAVVFGSFSV(14)DDHLMALVFRRFAATV-----LFPVFRVDGIGHSFVNNPLPLNTEATVVGPTRL(31)
Wolbachia sp. wMel            ......L.Q.HI...VH..I..D.VN( 2)N.N.VEV.KE...KS.-----N....TMK.V..GHT.D...F..H.IISVQDEK(12)
Wolbachia pipientis wPip      ......L.Q.HI...VD..I..D.VN( 2)N.N.VEI.KE...KS.-----N....TIK.V..GHR.D......HTIISMQDEK(10)
Orientia tsutsugamushi        .MR.E.L.Q.HIL.EVQ..I..DILC( 2).EQ.LE..KH...QS.-----N....TLN....ART.Y......R.VINHIKGN(15)
Rickettsia felis              D.E.L.L.Q..LLEDVK.II....GK( 0).LDATM..L.N..DNL-----NI...KTNRF..EKI.D.IIY..DSKIIMGKNK( 8)
Rickettsia bellii             D.A.IQL.Q..LLENIK..I..T.DK( 0).SK.TM..L.N..NNL-----KV...KT.RF..EKI.D.IIY..DSKII.NGEK( 7)
Sodalis glossinidius          D.T.HQLEYKKN.H-PK..I..Q.YS( 6)EKD.YRY.LQE..NR.-----DY..YYYPEF..GKT.Q.FI.GQQMQILCNEHY(12)
Francisella tularensis        D...HQLLYLPENKKPE.II..Q.YP( 6)QRLIYKT.IKK..K.F-----NR..YYFPF...GQY.K..L.GVTSNIKCSKET( 8)
Vibrio augustum               D.Y.QQLLFLKD..-VN.I...QYYN( 6).RLMYKH.LEQ..KQL-----GK..YYFPF...GKY.Q.VIFGRQVSLQRHELN(14)
Rickettsia felis              H.N.VQL.N..T.ECIE.II..D.TK( 0)G.EFVEQAIKS.CLNHIP---HIEAYKA.....GE..H.VIM.H.VIINSNVLS(11)
Rickettsia bellii             H.N.VQL.N..I...IE.II..D.TS( 0)G.ENIE.AIKS.CLNHIP---NIKTY.AE....GT..Q.VIM.H.VVIS.NYLS( 8)
Salmonella enterica           S.K.MGLAYSP.IKNCLG.I..DVIK( 9)VQSQFDYIIH..SEKFIG--DKIALKANNIF..GAI.M......F.VIKRNGAS(10)
Bdellovibrio bacteriovorus    D.MFEQFRQ..ILKKCHGI.L.D.IG( 6)TEDKTQ...K.W..DL-----EI.L.KGLEA..AP.QR.V.F..PCEL.MANNR(11)
Rickettsiella grylli          D.M.Q.LYQS.I.NHVK.I.L.N.TF( 5)EEEKIQTILK...IEQ-----HC..L.CPEV..GRK.RS..FGKQ.HLDLNKN.( 7)
Pseudomonas aeruginosa        ....WQLLESIDARQLG.ICL...TD( 5)VA.SLERI.GEY..AI-----EV.LYHHLPS..GAQ.RAW.YGKT.VLE.NRLR( 1)
Burkholderia cenocepacia      D..IMT.RH..IL...R.I.L.D.VR( 6)ANY.LTD.LLDVL.PL-----GA.IYGGLPV..GQR.LSWVVGRP.EIR.GILF( 1)
Bacteroides thetaiotaomicron  ..MMYNL..G..LEKLSGLII.Q.TE( 6)LGKELYPALADLVKEY-----DY..CFNFPV..VTH.L..INGAKVELTVGKKN( 7)
Cytophaga hutchinsonii        D.MMVQL.RS.KLKKLKGLIV.HMTD( 6)FGQSVTDIILSHTKEY-----T..ICFDFPA..EAP.M.VI.GLKSELIVAKSE( 6)
Anaeromyxobacter dehalogenan  D.Y.TQLR...AL..VAG.AL.Q.TE( 3)AGVRG.E.V.ELVSGL-----GV..AEGFPA..DDA.FSV..GAR..L.A.GPG(22)
Stigmatella aurantiaca        D.MWTQLR...L.QQVRGI.L.D.TA( 5)AEYTS.E.L.SL.QEE-----GL.CAAGFP...GAL.F.V..GVTVRLEADKAQ(10)
Crocosphaera watsonii         D.M.TQWR.L.A.S.VKGIAL.R..G( 5)NSWTVEE.L.DRLGDL-----GI.IVSELPF..DG..AI..VGMTVKLD.DRGS( 5)
Desulfotalea psychrophila     D.M.TQLAY..K.KDVAGILL.D...(11)HREMVWSRLLEIVGKR-----NI..LAGIRS..IMD.H..L.GSTVR.APGE.R( 9)
Bordetella bronchiseptica     D.M.AQLRR..A...VRG.LV...TR(10)AQAALYPLV.EHFQAR-----GI..LAGWPS..GDP.LT...GARV.LDAGRGA( 9)
Pseudomonas entomophila       D.L.TQLR...KLE.VRG.LV.D.AG( 1)TVASLTPLLLDIFGPL-----GV..LAGWRS..CNP.VC...GARVQLDAGQQR(10)
Streptomyces coelicolor       D.Y.TQLLRS.WL..VGG.LL..WAQ( 2)PYERLRPLLADRLGGL-----GV..VEDF.F..CEGALTV.FGVP.ELDAD.GT(12)
Symbiobacterium thermophil    D.M.VQLL...KLQ..AGIL..DSPT( 8)PSLTLPE.LTELLGPL-----GI..LYGFPC..GPHRAT...GVRTRLDAAGGT(10)
Clostridium beijerinckii      D.M.LQL.QC.K.KD.AGIIL..WTG( 7)NSLTLMEI.EELIKPE-----NT.TIYNLAC..CAPTISI..GARVKIN.ERSE( 4)
Clostridium novyi             D.M.SQLE.G.KLKDCSGFII.Q.TK( 7)RSLTLEEILEDKILSL-----GK.TISNFMS..DYPKLT..IGARGVINCNNNK( 9)
Bacillus licheniformis        D.M.NQL.M..KLSD.AGILLCD.HN( 7)KSLTLKE..QDYI.SE-----GK.ALSGFK...CSP.IAV.VGVK.VLDAGKKR( 8)
Bacillus cereus               D.M.NQLL.S.K.NECRG.I.T.CHD( 5)PSQSLQTILYEYF.PY-----HI..LFDLP...ISP.IGI..GAT..INTNNKT(13)
Sphingomonas sp. SKA58        D.M.TQL..G.IL.KLAG.A..QCTD( 8)GGFTVSE.LQQHL.PL-----GI.A.QGGQF..VANQYS...GIQ.EMDAT.GT( 9)
Anabaena variabilis           D.M.TQL.N..ILYRISGFI..QCTD( 6)KSQTLIE.LQDHITPL-----KI.SWYGAM...IQDKFI..IGANVEIDADAGT( 9)
Neisseria gonorrhoeae         ..M.NTLY.S.ILGKQR.I...D.RM( 8)SSYDFSA.AKHISR.A-----KI..LTGFPF..IADKITF..GAHTRIRMNGNG(41)
Escherichia coli LdcA         ..M.LQLYH..ILPRQK.IIL....G( 7)AGYNLES.YAFLRSRL-----SI.LITGLDF..EQRTVT...GAH.ILNNTREG(13)
Chlorobium ferrooxidans       D.M.S.LSN..LLARCG.LL..Q..S( 6)EAERVKNI.DYYSTLNP----NAT.LSGLSY..IRELMTI.IGARFR.SVTASG(13)
Thermoplasma acidophilum      D.Y.FT.....IL.KFEGFA..E.KS( 6)PMPFVEDIIEMYMNSL-----KKVSIYGLPF..GEDQMMI...ARVRISYEEPY( 9)
Ferroplasma acidarmanus       D.YFFS....HIIEQFNGF...D.KA( 6)PMPSIED.VQK.MNEI-----NK.SLYGAPF..GEEQMV....ARIGISDEEPY( 9)
Gibberella zeae               QAAFADLIAQ...EE.AGL.V.RPYG( 5).RETY.GIIKGLLCEGRLASKK..ILFNVD...TVPMLT..YDAL.ELDSE.GT( 9)
Haloarcula marismortui        GAN.RALGER.LLERFDG.LV.RACA(14)YRERQRDTIADILK.YNP---NA..VFNCEF..TYPTC.I.IGG.VEIEPA.KS( 4)
Bacillus cereus ATCC10987     ...FSLL.IN....KISGIIL.KHEQ( 5)TNRKPFEILLEVLQNQ-----RI.FLADFDCC.THPMITM.IGIQVKLDATNKT( 9)
Lactobacillus casei           RTY.E.LDEH..LAQVK.ILV.KPQN( 2)YFDAYQQ.LLDITQPY-----KT.ILYNLNF..AYPRTV..YGLQT..DFDQ.Q(12)
Bacillus cereus ATCC10987     KYW.RNYAAQ.ILQK.KGII..KPKD( 2)YYEEYKHEILQVMKEHNL--EDL.ILYNLNF..TEPKFI..YGSM.EIDCENGS( 9)
Escherichia coli MccF         ..LFSML..NR...KVS.IIL.KHEL( 5)SKRRPYE.LTEVLDGK-----QI..LDGFDCS.THPMLT...GVKLAIDFDNKN(12)
Consensus                                      O                                            *              O
```

Continued.

B

```
Acyrthosiphon pisum              ( 93)DDVFERGVCSYY-------KGPSITANGDVFDENEMTAAHRTLPFNTMVKVET----MGTSVVVKINDRKTAADGKVMLLSRAAAESL( 55)
Aphis gossypii                   ( 85)NS...K.T.A.-------Q.GIE....E..N........Q........N..I---.......R.....DDLN.HILDM.F....AV( 52)
Toxoptera citricida              ( 29)NN...T...A.--------..KIE....EI..D................N..I----..A....R.....DDSN.HILDMYL....GI(  0)
Sphingopyxis alaskensis          ( 70)TGHSFS.MA...G----NES.SR-..S.QR.NQ.A......S...G.KLR.TH----G.S..I.T....GPFIR.R.LD..TG..RAI( 16)
Bradyrhizobium sp. ORS278        ( 95)LQTASS.LA...S------E.HK-..S.ER..PS.L.....S...G.RLQ.TNVR--T.R....RV...GPFVQ.R.VDV.YS..QA.( 16)
Cytophaga hutchinsoni            (138)AGQSGQCLA.M.G------DPQP-..S.EI..PTQL....P....H...E.TNTA--T.R..T.R....GPYVA.RCLD..T..FSAI( 16)
Comamonas testosteroni           (146)VMETGSCGA...S------E.QL-..S.ES..PSA.....K....D...R.TNPV--TS...T.R....GPFIT.RCID..Q..FAVI( 16)
Polaromonas naphthalenivorans    (162)KSFT.T.YA.W.GGLKDGFH.KK-.....R.NR.LL....K...LPCL...TNKA--NNKA.ILMV...GPFKKNRIIDV.QR...I.( 73)
Pseudomonas fluorescens          ( 57).HYE.I.IA.W.G---EGFHNKL-.......SRFSL....K...MPSF.EITNLE--N.KK.I..V...GPFVENRIID..EK..IL.( 92)
Pseudomonas putida               ( 49)KHYE.I.IA.W.G---IEDH.TL-....EA.NRHLIS...K...LPCF.L.TNLE--N.RKL.TRV...GPFIE.RIID..EK..QV.( 93)
Chromohalobacter salexigens      ( 81)TKYKAE.LA.W.G---DAFH.RL-....E...MASLS...P...LPCY.R.TNLG--NSK.L..RV...GPYHGNRLIDV.NK...L.(116)
Salinibacter ruber               ( 87).GYD.T.IA.W.G---PNFH.RP-....ET..QHA.....T...IPSI.E.TNLE--N.R..I.RL...GPFV.NRLID......TE.(153)
Borrelia burgdorferi             ( 71).KYDAT.IA.W.G---PQFH.KQ-....EI..Q.A.....P...MPVLAR.TNLE--N.K.II.R....GPF.A.REID..KH...Q.(186)
Rickettsia conor                 ( 71)F.YD.T.IA.W.G---PDFH.KL-....E...Q.AVS...K...MPSV.R.TNLE--N.R.L..RV...GPF.H.R.IDM..RS.QL.(174)
Anaplasma marginale              (108)VPW..I..A.W.G---EPFH.RR-....E.Y.MHG.....K.M.LPSYAV.RNRV--N.RQ...RV...GPFI..R.ID..F...RK.( 40)
Wolbachia sp. wMel               ( 56)PRSV.T.LA.W.G---PGFE.RQ-....EI..STQL......MRLPSRAR.TRLD--T.A.II.RV...GPYI..RLID..H...QA.( 59)
Magnetospirillum gryphiswaldense (108)KPYK...QA.W.G---KRYH.QR-..S...Y.MYS.....P...IPSY.R.TNLQ--N.S..I.R....GPFLESRLID..YV..HK.(127)
Parvibaculum lavamentivorans     (122)ERYKT..MA.W.G---RRYH.QK-..S.E.Y.MYA.....P...IPSYAR.TSVT--S.K....RV...GPFLSNRLID..YT..YK.(190)
Maricaulis maris                 (124)RNYV...TA...G---AKFH.RL-.S.RE.Y.MYQF....K...LPSFAR.TNLD--N.E..I.RV...GPFH..R.VD..Y...VR.(217)
Nitrobacter winogradskyi         ( 95)RAYRMV.TA.W.G---TKFH.QA-....ETY.LYG.....K...LPSY.R.TNLD--N.K..I.RV...GPFYSDR.ID..F...KK.(164)
Nitrosospira multiformis         ( 55)AEFTQT.NA.W.G---GRFH.RK-.SG.ERY.M.AF....K...IPSY.R.TNTK--N.K..I.RV...GPFHGNRIIDV.K...QK.(102)
Nitrosomonas eutropha            (108)K.FR...IA.W.G---SKFH.HL-.S..E.Y.MYA.....K...IPSY...RNED--NNKEII.RV...GPFHE.R.ID..Y...YK.(107)
Neisseria meningi                ( 72)KGFT.S.KA.W.G---KKFH.HL-.S..EIY.MYS.....K...IPSY...TNTD--N.K.T..RV...GPFH..RIID..Y...YKI(109)
Pseudomonas aeruginosa           (163)AAFA.T.IAIV.G---DEFA.LP-....E..SQT.L....PS..LPSL.S.TNVD--T.RE...RV...GPFE..ANVQ...K...T.(165)
Vibrio cholerae                  ( 74)SRFSQA.LAAI.D---AEPGSNL-..S.EA..PTQL....P...IPSYARITNLA--N.RMI..R....GPYGNDR.IS......DR.(206)
Shewanella denitrificans         ( 71)QNFSQI.LAAW.G---EEAN.NT-..T.ET..P.AL....P...IPSY.R.TNVS--N.RQI..RV...GPYTS.R.ID..K...DR.(219)
Stenotrophomonas maltophilia     ( 46)VGDTAS.IA.W.G---PNFH.KK-.S..ESYNM.A.....K...M......SNLK--N.K.T..R....GPFVA.RIID..K...NDI(111)
Methylibium petroleiphilum       ( 78)R.GQ.T.IA.W.G---PTFY.KR-..S.AI.RK.AL........LG.R.R..NLE--N.R..D.V....GPFVR.R.ID..WK..NAI(103)
Hyphomonas neptunium             ( 51).CRY.V.YA.W.G---PKFHYRK-....ER.NMFKL...S..FSLG.Y.L.YNLE--N.KFT..R....GPYV..RIID..M...DEI( 38)
Yersinia mollaretii              (125)CPKYI..YA.W.G---GKFH.RK-..S.IR.NQH.YY....Y...G.LLE.KNLK--N.RK.I..VV..GPYVR.RHLD..Y...KK.( 19)
Escherichia coli                 (102)N.FKQE.TA.W.G---PGFH.KK-..S.EI.NQ..L....KR..LG.KIR.TNLK--N.K.........GPFHSNR.LD..Q...QKI( 16)
Oceanicaulis alexandrii          (113)S.FRQT.RA.W.G---KGFH.RR-....ER.NM..F........LASY.R.TNQA--N.K.........GPFSR.RILD..Y...KVI( 35)
Haemophilus influenzae           ( 80)S.FHQT.RA.W.G---RGFH.RR-....ERY.MHAL........LGSY.R.TNPA--TSR....R....GPY.R.R.ID..M...SA.( 38)
Microscilla marina               ( 40)LNSATV.LA.W.G---EAFH.KT-....EK..MMAL....KE.....T...TNLL--NNR....R....GPFRKDRIID..KY...K.(153)
Shewanella putrefaciens          ( 60)NTLVGE.KA.F.A---NQFH.RT-....ET.NM.KL....PS...G.W.R.TNMR--T.KD...R....GPFVK.RIID..IG..KE.( 25)
Salinispora tropica              ( 80)GLPDAQ.IA...A---DKFV.RT-....EIY.HQGL.....S...G.R.RTTRIDAPAQP....R....GPFK..RIID..K...GR.( 43)
Thermobifida fusca               ( 77)RESDQA.LA.W.G---EQFH.RK-..S.ER..SMD.....K....G.R.C.RSSV--T.K....R....GPF.P.R.ID..K...QE.( 66)
Erythrobacter litoralis          (113)ALEL...HA.W.G---GQFH.RR-..S.ENY.KYAL....K....G.I.R.RSLA--L.RE.D.R....GPF.P.R.IDV.Q....A.( 43)
Chlorobium ferrooxidans          ( 80)ARSLGM..A...G---RKFH.RR-....ER..M.G.....K....G.K.R.TNAS--N.R....R....GPFVR.RTID...K..GD.( 16)
Campylobacter fetus              (241)IQFQFQ.WA.W.G---PGFH.RL-S...ER.NQYA.....K....G.K.R.TNLH--N.S..I.R....GPFIP.R.ID..S...DV.( 19)
Aquifex aeolicus                 (233)VTSRRK.MA.W.G---PGFH.RL-....ERYNQ.GL....K....G.Q.R.TNLR--N.R..I.R....GPFVG.R.ID..KG..SVI( 17)
Burkholderia phytofirmans        (223)IKRIKG.IA.W.G---PGFH.RR-....ER.NQ.AL.........G.R...TNLR--N.Q....R....GPFTG.R.ID..AG..RAI( 16)
Burkholderia mallei              ( 16)GATS...QA...H---DRYQ.QR-..S.EPY.AQAL.....R...D.R.R.TNLD--N.RQ.T.R....GPFTE.R.ID...R...R.( 16)
Leptospirillum sp Group II UBA   ( 25)RGYDQT.TA...G---SRHH.KR-..S.EP.NQHAL.....S...G.R.R.TNMA--NQR....R....GPHTR.RLID.......KI( 17)
Dichelobacter nodosus            ( 25)HGYDQT..A...G---AKHH.KR-..S.EA.NQ.SL.....Q...G.R...TNLK--NDE....R....GPHTR.RLIDV......Q.( 17)
Bradyrhizobium japonicum         ( 39)VGFT.T.QA.F.G---DKHQNKQ-..S.ELYKHKLK....KK...GSS...TNVK--N.K..I......GPFVR.RIID..KS.FS.I( 16)
Trichodesmium erythraeum         ( 13)KNFIQ..IA...T---DRFD.RK-..S.EI..NR.LVGS.KK.........TNLA--NKR....R....GPY.H.RIIDV.KS..NLI(153)
Crocosphaera watsonii            ( 65)KSYIKE.LA...H---LKFD.RK-..S...YNSKQF....K...I.SYAL.TNLH--NNRK.I.R....GPFS.KRLID..H...KEI(140)
Synechocystis sp. PCC 6803       ( 34)ETEIVG..A...G---NELA.NR-..S.ER..PGQL.........GS..R.TNVA--T.D....R....GPF.H.R.IDV.H...REI( 18)
Haemophilus ducreyi              ( 39)KSEPTT.SA...A---NKFQ.KR-..S.ETY.K.KF......YA.G.KL..TNIK--T.IWIQ.YV...GPY.KDRIIDV.Y...TA.(128)
Actinobacillus pleuropneumoniae  ( 77)RSHYQS..A...A---DKFN.RR-....ER.SNSA.....K....G.L.E.TNTR--N.R....RV...GPY.HAR.VD..K...RQ.( 28)
Aquifex aeolicus                 ( 72)RSHYQS.IA...G---DQFN.RL-....EL.SN.......K....G.LLE.TNVR--N.R..I.RV...GPYYGSRIVD..K...IE.( 30)
Consensus                                        *            OO    O      OO                          O **        O O   O   O
```

Continued.

The GenBank accession numbers of sequences used here are as follows: (A) LD-carboxypeptidase. *Acyrthosiphon pisum*, AB435382; *Wolbachia* sp. *w*Mel, NP_966741; *Wolbachia pipientis* wPip, YP_001975416; *Orientia tsutsugamushi*, YP_001248242; *Rickettsia felis*, YP_246639; *Rickettsia bellii*, YP_537751; *Sodalis glossinidius*, YP_455202; *Francisella tularensis*, YP_169172; *Vibrio augustum*, ZP_01235509; *Rickettsia felis*, YP_246832; *Rickettsia bellii*, YP_537514; *Salmonella enterica*, YP_216231; *Bdellovibrio bacteriovorus*, NP_968807; *Rickettsiella grylli*, ZP_01300534; *Pseudomonas aeruginosa*, NP_253885; *Burkholderia cenocepacia*, EAY65659; *Bacteroides thetaiotaomicron*, AAO77656; *Cytophaga hutchinsonii* ATCC 33406, YP_679995; *Anaeromyxobacter dehalogenan*, YP_466599; *Stigmatella aurantiaca*, ZP_01461884; *Crocosphaera watsonii*, ZP_00518654; *Desulfotalea psychrophila*, YP_066013; *Bordetella bronchiseptica*, NP_889214; *Pseudomonas entomophila*, YP_610257; *Streptomyces coelicolor*, CAA22737; *Symbiobacterium thermophil*, YP_076637; *Clostridium beijerinckii*, YP_001309644; *Clostridium novyi*, YP_879234; *Bacillus licheniformis*, YP_078586; *Bacillus cereus* ATCC14579, NP_831148; *Sphingomonas* sp. SKA58, ZP_01304762; *Anabaena variabilis*, YP_325418; *Neisseria gonorrhoeae*, YP_208343; *Escherichia coli* LdcA, NP_415710; *Chlorobium ferrooxidans*, ZP_01386568; *Thermoplasma acidophilum*, NP_393714; *Ferroplasma acidarmanus*, ZP_01708781; *Gibberella zeae*, EAA73228; *Haloarcula marismortui*, AAV46771; *Bacillus cereus* ATCC10987, NP_979581; *Lactobacillus casei*, ZP_00386368; *Bacillus cereus* ATCC10987, NP_978349; *Escherichia coli* MccF, CAA40814. (B) rare lipoprotein A. *Acyrthosiphon pisum*, AB435384; *Aphis gossypii*, DR391796; *Toxoptera citricida*, CD450666; *Sphingopyxis alaskensis*, YP_618011; *Bradyrhizobium* sp. ORS278, YP_001204245; *Cytophaga hutchinsoni*, ZP_00309644; *Comamonas testosteroni*, ZP_01522477; *Polaromonas naphthalenivorans*, YP_980734; *Pseudomonas fluorescens*, YP_346566; *Pseudomonas putida*, NP_743090; *Chromohalobacter salexigens*, YP_573561; *Salinibacter ruber*, YP_446246; *Borrelia burgdorferi*, AAC67080; *Rickettsia conor*, NP_360174; *Anaplasma marginale*, YP_153851; *Wolbachia* sp *w*Mel, NP_966279; *Magnetospirillum gryphiswaldense*, CAM76691; *Parvibaculum lavamentivorans*, YP_001414372; *Maricaulis maris*, YP_756686; *Nitrobacter winogradskyi*, ABA04727; *Nitrosospira multiformis*, ABB73623; *Nitrosomonas eutropha*, ZP_00669959; *Neisseria meningi*, AAF40721; *Pseudomonas aeruginosa*, ZP_00205119; *Vibrio cholerae*, AAF94110; *Shewanella frigidimarina*, ZP_00639800; *Stenotrophomonas maltophilia*, ZP_01645980; *Methylibium petroleiphilum*, YP_001022529; *Hyphomonas neptunium*, YP_760519; *Yersinia mollaretii*, ZP_00825993; *Escherichia coli*, NP_415166; *Oceanicaulis alexandrii*, ZP_00951821; *Haemophilus influenzae*, NP_438203; *Microscilla marina*, ZP_01694079; *Shewanella putrefaciens*, ZP_00815483; *Salinispora tropica*, YP_001156950; *Thermobifida fusca*, YP_289586; *Erythrobacter litoralis*, YP_458460; *Chlorobium ferrooxidans*, ZP_01385536; *Campylobacter fetus*, YP_892348; *Aquifex aeolicus*, NP_213946; *Burkholderia phytofirmans*, ZP_01510848; *Burkholderia mallei*, YP_104418; *Leptospirillum* sp Group II UBA, EAY55979; *Dichelobacter nodosus*, YP_001209850; *Bradyrhizobium japonicum*, NP_767084; *Trichodesmium erythraeum*, YP_722449; *Crocosphaera watsonii*, ZP_00513775; *Synechocystis* sp. PCC 6803, BAA10397; *Haemophilus ducreyi*, NP_874337; *Actinobacillus pleuropneumoniae*, ZP_00133774; *Aquifex aeolicus*, NP_213799.
